# Effect of Lubricating Phase on Microstructure and Properties of Cu–Fe Friction Materials

**DOI:** 10.3390/ma12020313

**Published:** 2019-01-20

**Authors:** Xiaoyang Wang, Hongqiang Ru

**Affiliations:** 1 School of Materials Science and Engineering, Northeastern University, Shenyang 110819, China; wxy927@163.com; 2 Key Laboratory of Advanced Materials Technology of Liaoning Province, Shenyang University, Shenyang 110044, China

**Keywords:** lubricating phase, powder metallurgy, friction material, microstructure, tribological properties

## Abstract

Cu–Fe-based friction materials with flake graphite, granulated carbon black, and high-strength graphite as lubricating phase were prepared by the powder metallurgy method. The effects of different types and mass fraction of lubricating phase on the microstructure, mechanical properties, and tribological properties were investigated. The results show that when the mass fraction of granulated carbon black is 5 wt%, it is easy to form a good interface with the matrix, but the interface is prone to pores and cracks when its mass fraction is 10 wt%. The bending strength and compressive strength properties of the composites increased with increasing in the mass fraction of granulated carbon black and reached the maximum of 40 MPa and 70 MPa at 5 wt% granulated carbon black, after which bending strength and compressive strength all decreased. The friction coefficient and the wear loss of the materials initially decreased as the mass fraction of granulated carbon black increased and obtained minimum of 0.436 and 0.145 mm when the mass fraction of granulated carbon black was 5 wt%, then ascended. Compared with the sample with 5 wt% high-strength graphite as lubricating phase, the sample with 5 wt% granulated carbon black as lubricating phase had better sintering performance, mechanical properties, and tribological properties.

## 1. Introduction

With the rapid development of high-speed railways, powder metallurgy friction material has been widely used. Powder metallurgy friction material exhibits many advantages, such as wear resistance, anti-bite, adversity to thermal cracking, and long life [1,2,3,4,5]. Powder metallurgy friction materials are mainly composed of a matrix, friction phase, and lubricating phase. In the lubricating phase, lamellar graphite (mainly natural flake graphite) has been the most widely used. Flake graphite has a layered hexagonal structure, with carbon atoms in the atomic layer and a strong covalent bond, but the atomic layer is connected by the weak van der Waals force, and when subjected to shear force is prone to slip. During the friction process, the graphite-rich layer formed on the friction surface prevents the direct contact between the metals, effectively reducing the friction coefficient and the wear of the material. 

It is reported that the use of natural flake graphite together with artificial graphite has a great influence on the friction properties [6,7,8,9,10,11,12,13,14,15]. Carbon black [16] and graphite have the same lubricity, and the advantages of carbon black are reflected in its wide range of sources and low prices. It is used as filler, mainly in rubber, polyamide, and other materials [17,18,19,20,21,22,23,24]. However, carbon black cannot be directly used as a lubricating phase in friction materials. The main problem is that carbon black has a large specific surface area and tends to agglomerate, affecting the moldability and sinterability of the material. In order to solve this problem, carbon black powder was granulated, which improved the sintering performance of the material. However, little has been reported so far on the influence of granulated carbon black and natural flake graphite mixed composition on the lubricating phase of the mechanical and tribological properties of Cu–Fe friction materials. Accordingly, in this paper, a friction material with different mass fraction of granulated carbon black and natural flake graphite as lubricating phase was prepared and compared with those of high strength graphite and natural flake graphite as lubricating phase. Meanwhile, the effect of the mass fraction and type of lubricating phase on the properties of the material is discussed in detail. The friction materials prepared in this paper are mainly applied to high-speed train brake pads and the use of low-cost granulated carbon black as a lubricating phase can reduce material costs and improve economic efficiency. By improving the manufacturability of materials, economic benefits can be improved, as several authors have reported [25,26,27]. The results of this study can be used as a reference for the popularization and application of Cu–Fe friction materials, and granulated carbon black is proved to be a suitable lubricating phase for the fabrication of friction materials.

## 2. Materials and Methods 

### 2.1. Raw Materials 

The experimental raw materials used mainly include: electrolytic copper powder (particle size <75 μm, purity ≥99.8%), water atomized iron powder (particle size <75 μm, purity ≥99.8%), ferrochrome (particle size <200 μm, purity ≥98%), natural flake graphite (particle size 100–600 μm, purity ≥98%), high-strength graphite (purity ≥98%), and carbon black powder (particle size <100 nm, purity ≥99%). 

### 2.2. Lubricating Phase Preparation

Phenolic resin with 75% mass fraction and nano-carbon black powder were uniformly mixed and left to stand for 12 h, and then cold pressed at 100 MPa for 1 min, followed by drying in the oven (Shenyang Sitong Electric Furnace Factory, Shenyang, China) at 50 °C for 12 h, and then placed in the muffle furnace (Shenyang Sitong Electric Furnace Factory, Shenyang, China) at 175 °C for 2 h. Granulated carbon black pacticles were obtained by crushing and sieving the carbon black bulk (GCB). The high-strength graphite (HSG) was obtained by crushing and sieving the commercial products. 

### 2.3. Friction Materials Preparation

The compositions of composites are shown in Table 1. The components were mixed by using glycerol and cold pressed at 200 MPa. The composites were then obtained by hot-pressing technique. The mixtures were heated to 600 °C and held at 25 MPa for 15 min in air. The specimens were cooled with the mold to room temperature and cut into preset sizes for mechanical properties and tribological properties tests.

### 2.4. Characterization

The microstructure and worn surface were examined and analyzed using scanning electron microscopy (SEM, Hitachi S4800, Hitachi High-Technologies Corporation, Tokyo, Japan) with energy dispersive X-ray spectroscopy (EDS, Horiba X-max, Hitachi High-Technologies Corporation, Tokyo, Japan) and laser scanning confocal microscopy (LSCM, Olympus LEXT OLS3000, Olympus Corporation, Tokyo, Japan). X-ray diffraction (XRD, PANalytical X’Pert PRO, PANalytical Company, Almelo, Netherlands) analysis was used for determining the phase of the worn surface. The porosities and densities were measured by Archimedes’ method. The bending strength and compressive strength were tested using an electronic universal testing machine (SANS CMT5505, MTS Systems Corporation, Shenzhen, China) under a constant crosshead speed of 0.5 mm·min^−1^. The tribological properties were evaluated using a disk-on-disk type laboratory scale dynamometer (MM1000-Ⅱ, Xi’an Shuntong Institute of Electromechanical Applied Technology, Xi’an, China). The rotor specimen was accelerated to a certain speed. The stator was loaded with a pressure against the rotor until the rotor was completely stopped. In this test, the moment of inertia was 0.82 kg·m^2^, the pressure was 0.5 MPa, braking speed were at 60 km·h^−1^, 120 km·h^−1^, 180 km·h^−1^, and 250 km·h^−1^, respectively. The wear loss was measured by micrometer with accuracy of 0.01 mm after brake at each braking speed 5 times. The temperature induced in the braking test was measured with a thermocouple located 1 mm behind the worn surface of the stator disk. 

## 3. Results and Discussion

### 3.1. Microstructures of Materials

**Figure 1:**
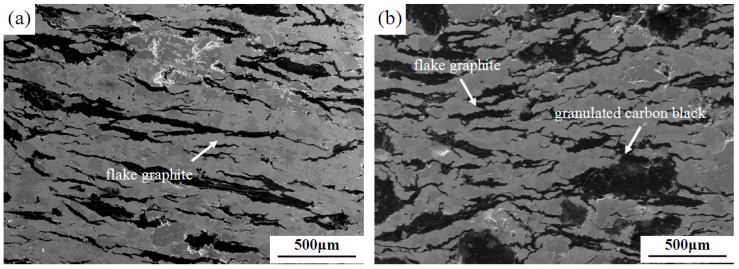

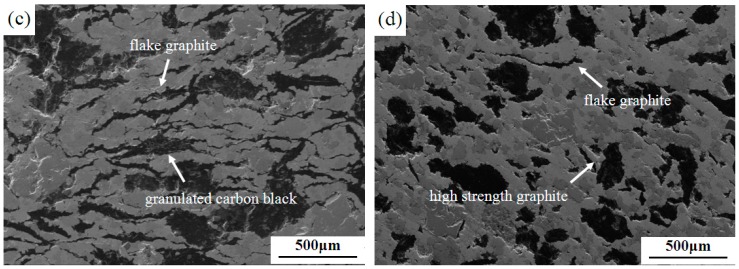
Microstructures with different lubricating phase: (**a**) 0% GCB; (**b**) 5% GCB; (**c**) 10% GCB and (**d**) 5% HSG.

Figure 1 shows the microstructures of materials with different lubricating phase in the direction parallel to the pressure direction. The elongated strips in Figure 1a–d are natural flake graphite, the wide strips in Figure 1b,c are granulated carbon black, and the approximate spherical shape in Figure 1d is high-strength graphite. When the mass fraction of granulated carbon black is 0 wt%, that is, all the flake graphite is added (Figure 1a). The flake graphite tends to be compressed into a flat strip shape and join with each other. In the case of granulated carbon black as the lubricating phase (Figure 1b,c), the granulated carbon black is deformed to form strip structures in this direction. In the samples with high-strength graphite as the lubricating phase (Figure 1d), the high-strength graphite no-strip morphology was observed. In the pressing pressure, the natural flake graphite produced a slip, and granulated carbon black were suppressed into deformation. High-strength graphite is artificial graphite; it has a certain strength to avoid significant deformation under pressure. Therefore, the morphology shown in Figure 1 is exhibited.

**Figure 2:**
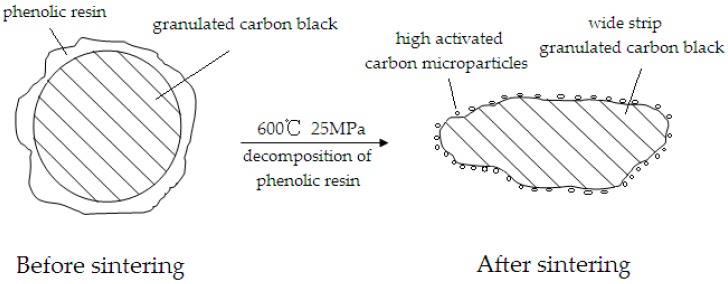
Schematic diagram of the lubricating phase structure change process.

The wetting property between the components of the material has a great influence on its microstructure and properties, and some scholars have conducted in-depth research on it. B.B. Straumal et al. [28] observed for the first time that other WC/WC grain boundaries are not partially wetted and are therefore “dry”. They are pseudopartially wetted, namely they have a high contact angle with cobalt binder, and nevertheless contain a 2–3 nm thin uniform Co-rich layer. In the majority of cases, the direct transition occurs from partial into complete wetting [29,30,31]. The sequence of discontinuous partial wetting to pseudopartial wetting and continuous pseudopartial wetting to complete wetting transitions wear observed in other articles [32,33,34,35]. A.B. Straumal et al. [36] considered the phenomenon of apparently complete GB wetting by a liquid phase. However, the wetting phase can also be solid [37] or even amorphous [38,39]. The wet ability between copper and graphite is poor, usually modified on the surface of graphite to improve wettability [13,40,41]. In the preparation of the lubricating phase, the surface of the carbon black is coated with a phenolic resin, and then subjected to high temperature and high-pressure treatment (600 °C, 25 MPa), and the carbon black is changed from a granular shape to a wide-strip shape. The decomposition of the phenolic resin in situ produces carbon particles of extremely small particle size. The carbon microparticles have extremely high activity and are easily diffused to the interstitial site of the Cu–Fe alloy, thereby increasing the interfacial bonding force between the lubricating phase and the metal matrix, which is equivalent to the wetting effect. The lubricating phase structure change diagram is shown in Figure 2. Therefore, it is necessary to further discuss the interface between the lubricating phase and the metal matrix.

**Figure 3:**
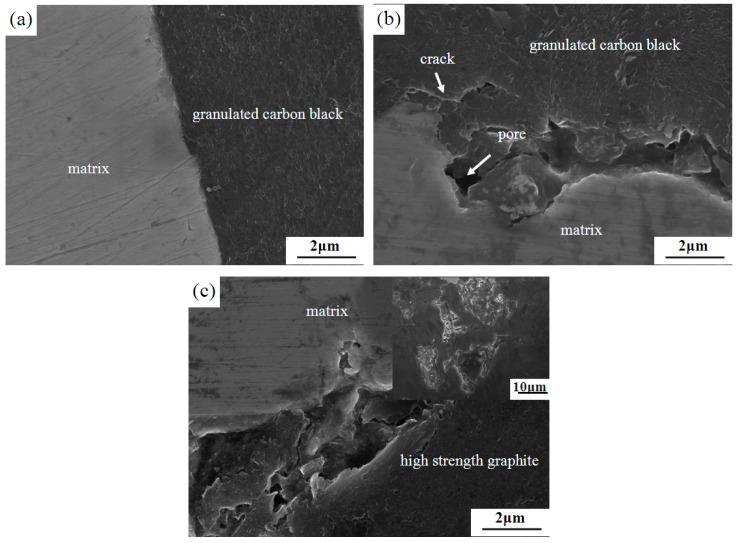
Interface microstructures with different lubricating phase: (**a**) 5% GCB; (**b**) 10% GCB, and (**c**) 5% HSG.

Figure 3 shows the interface microstructures of different lubricating phase materials. When the flake graphite and the granulated carbon black were used together as the lubricating phase, the mass fraction of the granulated carbon black had a significant effect on the microstructure (compared to Figure 3a,b). When the mass fraction of granulated carbon black is 5 wt% (Figure 3a), there was no gap between the metal matrix and the granulated carbon black, and the interface was well bonded. The granulated carbon black itself was compact with no obvious defects. In the case of 10 wt% granulated carbon black as the lubricating phase (Figure 3b), it was observed that there was a significant gap between the metal matrix and the granulated carbon black interface, the broken phenomenon appeared in the interface, and pores and cracks were present in the granulated carbon black. This phenomenon is related to the formation of agglomeration of too much granulated carbon black. Granulated carbon black with phenolic resin as a binder for granulation, and it belongs to the soft particles. Under pressing pressure, since the granulated carbon black is prone to deformation, it tends to form a complete interface with the metal matrix. In the sintering process, accompanied by pyrolysis and carbonization of phenolic resin, a large amount of gas is discharged. When the mass fraction of granulated carbon black is lower (5 wt%), less gas can be discharged in time, which does not affect the intact interface between granulated carbon black and metal matrix. On the contrary, when the mass fraction of granulated carbon black is higher (10 wt%), the gas is increased and trapped in the interface between the granulated carbon black and the metal matrix, which easily causes cracks and pores at the interface. 

Meanwhile, the 5% HSG sample (Figure 3c) exhibited an interfacial morphology similar to that of the 10% GCB sample. Compared with the soft granulated carbon black, the high-strength graphite and the metal matrix do not easily form a good interface due to the poor synergetic deformation ability.

In summary, the lubricated phases in 0% GCB (Figure 1a) and 5% HSG (Figure 1d) were untreated and did not have a wetting effect. The addition of granulated carbon black as a lubricating phase in 5% GCB and 10% GCB (Figure 1b,c) samples can provide a wetting effect on the bonding interface. However, the results show that the interface of 10% GCB is not well bonded, mainly due to the excessive content of phenolic resin, which produces a large number of pores during the sintering process, and the partial wetting effect is invalid. The effect of this phenomenon on material properties is discussed in detail below.

### 3.2. Sintering and Mechanical Properties of Materials

The sintering and mechanical properties of different lubricating phase materials are listed in Table 2. 

When the lubricating phase is completely flake graphite, micro-lamellar graphite cross-support each other, is not conducive to molding, resulting in low density of green body, so it has a higher porosity and lower strength (0% GCB). When the flake graphite was mixed with the 5 wt% granulated carbon black as the lubricating phase, the porosity decreased obviously, and the bending strength and compressive strength were increased significantly (compared with 0% GCB and 5% GCB in Table 2). Granulated carbon black is a soft particles; the deformation process can alleviate and absorb the deformation stress of the material, which can significantly improve the mechanical properties of materials. However, when the addition of the granulated carbon black increased from 5 wt% to 10 wt%, it was evident that the porosity of the material was increased accompanied by a decrease in the mechanical properties (compared with 5% GCB and 10% GCB samples). The sintering properties and mechanical properties of the materials are closely related to their microstructures and structures. From the above microstructure analysis, it can be seen that with the increase of the mass fraction of the granulated carbon black, the amount of gas to be discharged increases, the porosity increases, and an interfacial crack is liable to occur. Therefore, the mass fraction of the granulated carbon black cannot be excessive.

When the flake graphite and the high-strength graphite were used as the lubricating phase, the 5% HSG had lower density and higher porosity. Compared with 5% HSG, 5% GCB had better sintering performance and mechanical properties. Granulated carbon black belongs to soft particles. During the process of deformation, the soft particles have strong synergistic deformation ability, which makes it is easy to form a good interface with the metal matrix and can relieve and absorb the deformation stress of the material. Therefore, the porosity of the material can be reduced, and the mechanical properties of the material can be improved. In the pre-press and hot-pressing sintering process, high-strength graphite is not prone to deformation, its interface with the metal matrix is poor, and there are a lot of pores in the material to reduce the effective load-bearing area, resulting in lower strength of the material.

### 3.3. Worn Surface Analysis 

**Figure 4:**
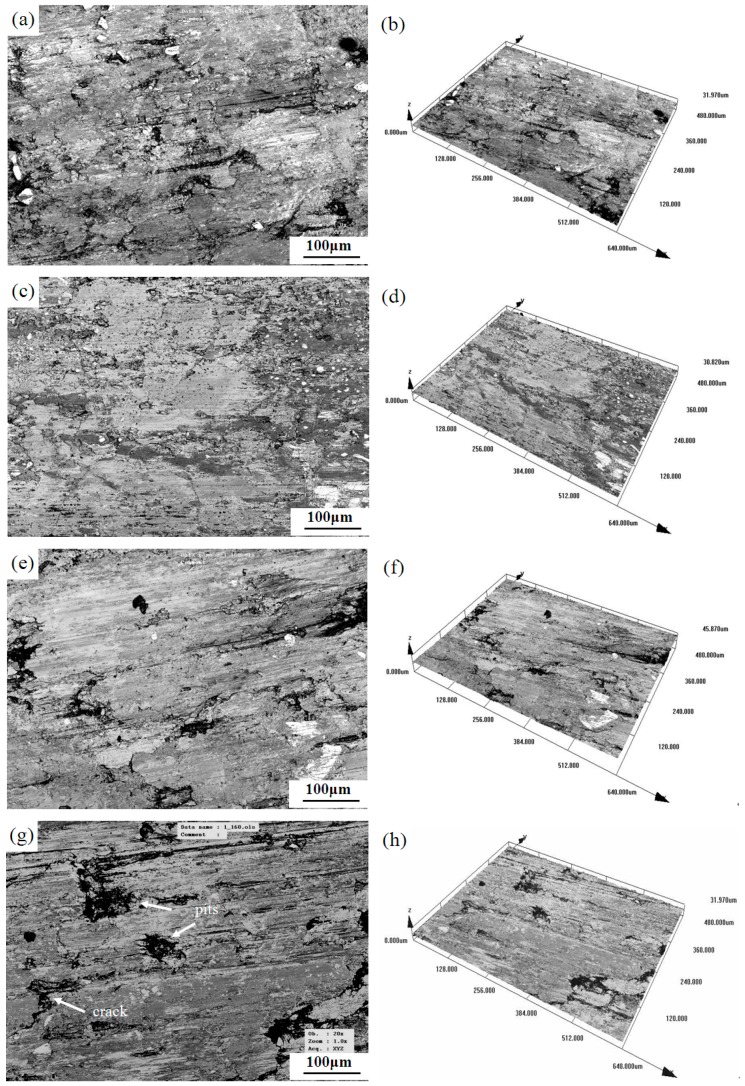
Worn surfaces with different lubricating phase: (**a**–**b**) 0% GCB; (**c**–**d**) 5% GCB; (**e**–**f**) 10% GCB; and (**g**–**h**) 5% HSG.

The LSCM morphology of the samples with different lubricating phase for worn surface is shown in Figure 4. When the mass fraction of granulated carbon black is 0 wt% (Figure 4a,b), the worn surface of the material is cracked and part of the surface film is peeled off, showing clear and deep grooves. A relatively flat and continuous film was formed on the surface of the sample with 5 wt% granulated carbon black (Figure 4c,d) as the lubricating phase, and the ploughing depth was light. 

When the mass fraction of granulated carbon black is 10 wt% (Figure 4e,f), the worn surface of the material is severely damaged. In the localized condition, there is serious adhesive wear and surface layer accumulation, resulting in inhomogeneous distribution and large depth of the grooves. When the mass fraction of granulated carbon black is 0 wt%, the deep grooves appeared on the friction surface and part of the surface film was peeled off; it is mainly due to the high porosity, low density, low compressive strength, and weak resistance to deformation of the 0% GCB in Table 2. When the mass fraction of granulated carbon black is 5 wt%, the porosity decreased and the resistance to deformation increased, and the surface film can maintain a relatively long time without being damaged due to the proper strength and less defects (5% GCB in Table 2). When the mass fraction of granulated carbon black is 10 wt%, the porosity increases, and more defects appear in the material (10% GCB in Table 2). In the friction process, defects in the surface film act as a crack source, accelerating the destruction of the surface film. With the rapid destruction and formation of the surface film, the groove depth increases instead. The sample with 5 wt% high-strength graphite has higher porosity, so the interface between the high-strength graphite and metal matrix is poor. In the friction process, high strength graphite is easy to fall off and break up, resulting in the formation of pits and other defects on the friction surface. These defects as the source of cracks accelerate the destruction of the surface film., thus forming a rough discontinuous surface film on the frictional surface (Figure 4g,h). 

**Figure 5:**
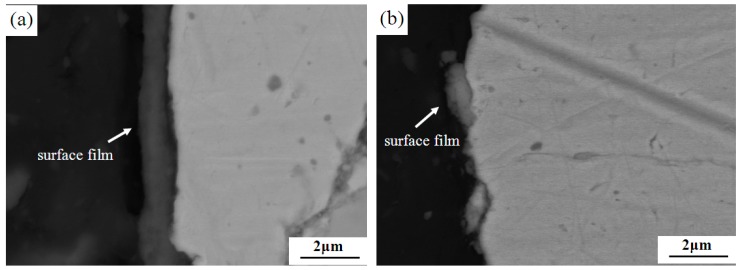
Morphologies of subsurface with different lubricating phase: (**a**) 5% GCB and (**b**) 5% HSG.

The surface morphology of the film has a very important relationship with the braking behavior of the material. In order to further investigate the case of the friction surface film, a comparative observation of the profiles of 5% GCB and 5% HSG samples were carried out, and the results are shown in Figure 5. The material with the 5 wt% granulated carbon black as the lubricating phase forms a friction film having a thickness of about 1 μm which is preferably bonded to the substrate on the surface (Figure 5a). The friction film of 5% HSG sample was peeled off and crushed without formation of a relatively complete and continuous surface film (Figure 5b), which is further confirmed with the analysis result in Figure 4.

**Figure 6:**
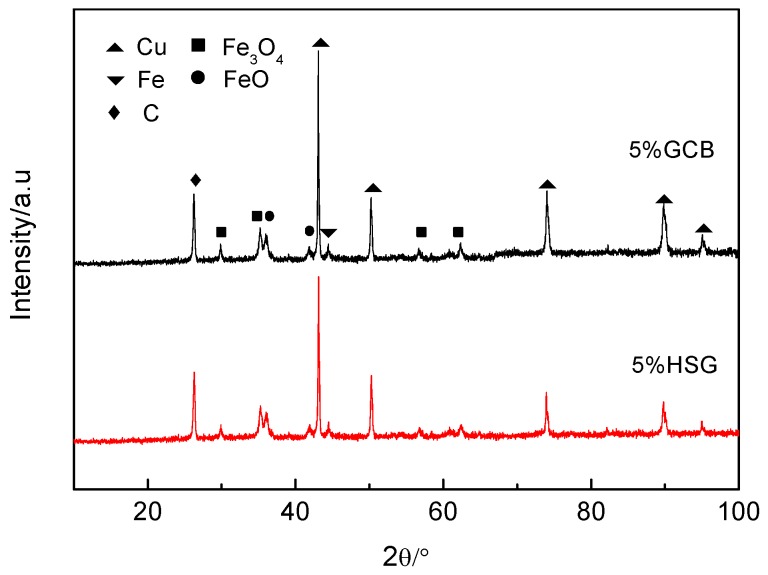
X-ray Diffraction (XRD) patterns of worn surfaces with different lubricating phases.

Figure 6 shows the XRD patterns of worn surfaces with different lubricating phases. During the friction process, the oxidation wear occurred on the surface of both materials. The diffraction peaks of FeO and Fe_3_O_4_ appeared, and the diffraction peak intensity of the 5 wt% granulated carbon black as the lubricating phase sample was slightly higher. A large amount of heat is generated on the friction surface during the friction process. As the friction process progresses, the freshly worn surface reacts with oxygen in the atmosphere to form an oxide film on the surface. The friction coefficient of the material can be effectively reduced by the existence of the oxide film. The higher intensity of the diffraction peak of the sample with the granulated carbon black as the lubrication component may be related to the formation of a more complete friction surface film.

During high-speed train braking, the surface temperature of the brake pad material rises rapidly. Excessive temperature affects the integrity of the surface film, while a relatively complete surface film can improve the friction properties of the material. Some scholars have suggested that using a cryogenic and minimum quantity of lubrication techniques in mechanical processing can significantly improve tool life, cutting speed, cutting forces, and surface integrity [42]. In this paper, the surface film integrity of the 5% GCB sample is better than that of the other samples (Figure 5 and Figure 6), which is beneficial to obtain a better performance of the friction brake material.

### 3.4. Effect of Mass Fraction of Granulated Carbon Black on Friction Coefficient of Materials

**Figure 7:**
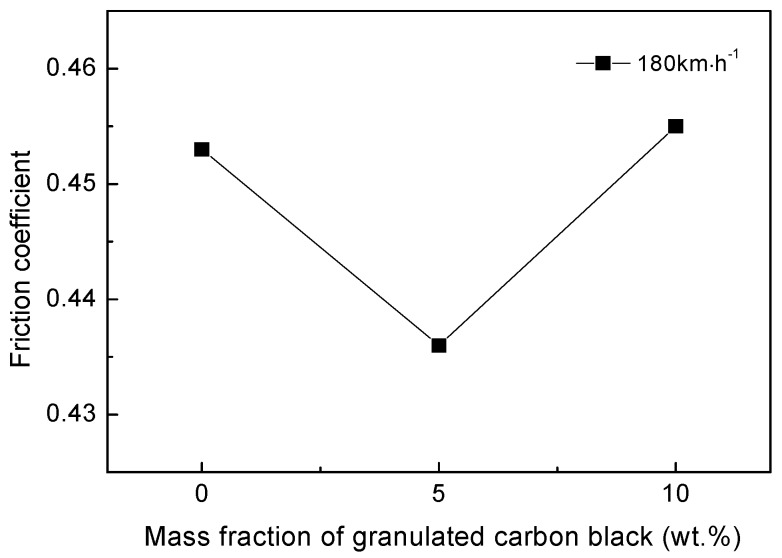
Effect of mass fraction of granulated carbon black on friction coefficient of materials.

The friction coefficient of the material varies with the mass fraction of the granulated carbon black as shown in Figure 7. When the mass fraction of granulated carbon black is 0 wt%, the friction coefficient is higher due to the higher porosity, weak surface film and matrix bonding force, and higher surface roughness. With the increase of the mass fraction of the granulated carbon black, the friction coefficient decreased. When the mass fraction of granulated carbon black is 5 wt%, the porosity is low, and the surface film is relatively smooth, so the friction coefficient of the material is the lowest, which indicates that the addition of granulated carbon black as lubricating phase can reduce its friction coefficient. On the contrary, when the granulated carbon black was added in excess (10 wt%), the porosity was increased, and the surface film was easily peeled off, resulting in an increase in the friction coefficient of the material.

### 3.5. Effect of Different Lubricating Phase on Friction Coefficient of Materials

**Figure 8:**
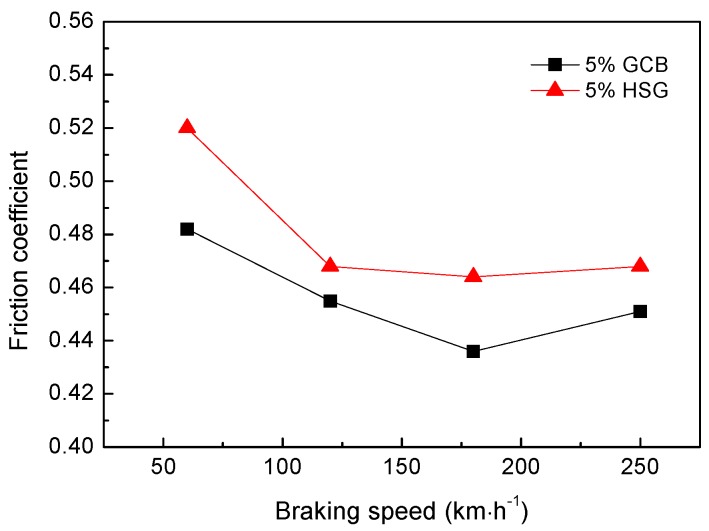
Effect of different lubricating phase on friction coefficient of materials.

**Figure 9:**
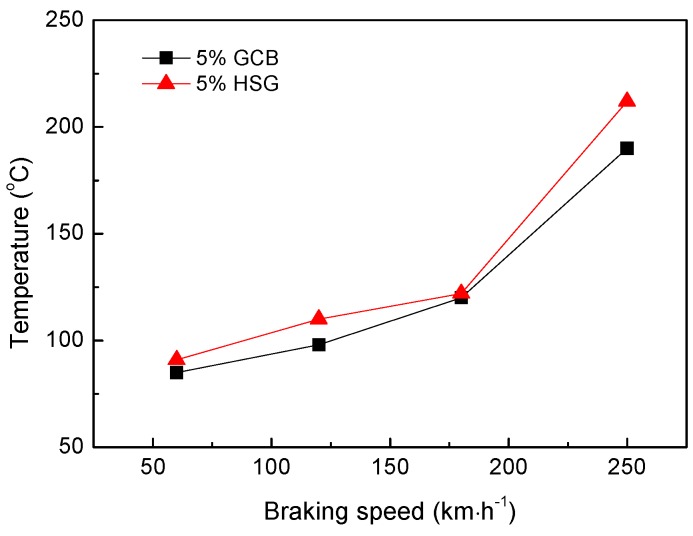
Effect of different lubricating phase on worn surface temperature of materials.

Figure 8 shows the effect of different lubricating phases on the friction coefficient of materials under the same mass fraction (5 wt%). The friction coefficient of 5% GCB is lower than that of 5% HSG. With the increase of the braking speed, the friction coefficient of the composites decreased, the friction coefficient reached the minimum value at 180 km·h^−1^, and then increased slightly at 250 km·h^−1^ instead. The coefficient of friction of the materials is between 0.43 and 0.52. The friction coefficient of the material is related to the braking condition and the friction surface morphology. The surface film of the sample 5% GCB is better bonded to the matrix (described in Section 3.3), which is easier to maintain relatively stable in friction, while the smooth continuous friction surface film reduces the coefficient of friction of the material. The 5% HSG formed a rough surface and a discontinuous surface film after friction (Figure 5c), resulting in a higher coefficient of friction of the material. As the braking speed increases, the friction power increases causing the sample surface temperature to rise. Because the thermal expansion coefficient of the surface film is different from that of the matrix, the surface film is prone to crack and break under the effect of thermal stress, so the rough surface is formed during the friction process, which increases the friction coefficient of the material. The curves of the worn surface temperature of the materials with different lubricating phases vary with the braking speed as shown in Figure 9. When the braking speed is lower than 180 km·h^−1^, the surface temperature of the material is not high enough to make the friction surface film and the matrix thermal mismatch; the friction surface film can be stable for a period of time, so that the friction coefficient of the material is reduced. When the braking speed is higher than 180 km·h^−1^, the surface temperature of the material rises sharply, and the surface film is easily destroyed by the thermal stress, resulting in a slight increase of friction coefficient at 250 km·h^−1^. This is consistent with the results in Figure 6.

### 3.6. Effect of Mass Fraction of Granulated Carbon Black on Wear Loss of Materials

**Figure 10:**
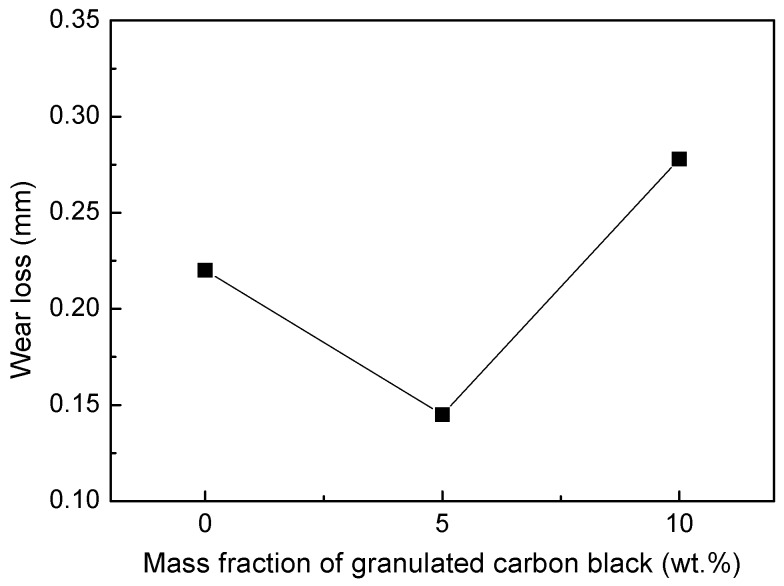
Effect of mass fraction of granulated carbon black on wear loss of materials.

Figure 10 shows the effect of the mass fraction of granulated carbon black on wear loss of materials. The trend of the wear loss of the material firstly decreases and then increases, and when the mass fraction of the granulated carbon black is 5 wt%, the wear loss of the material reaches the minimum value. Wear of the material is related to its mechanical properties, friction coefficient, and microstructure. Material has good mechanical properties and low coefficient of friction, accompanied by low wear loss on the material. The variation rule of the wear loss of the material is consistent with the law reflected in Table 2, Figure 3, and Figure 7. The wear loss of the 5% GCB sample is the lowest because of the best interface, the highest mechanical properties, and the lowest friction coefficient.

### 3.7. Effect of Different Lubricating Phase on Wear Loss of Materials

**Figure 11:**
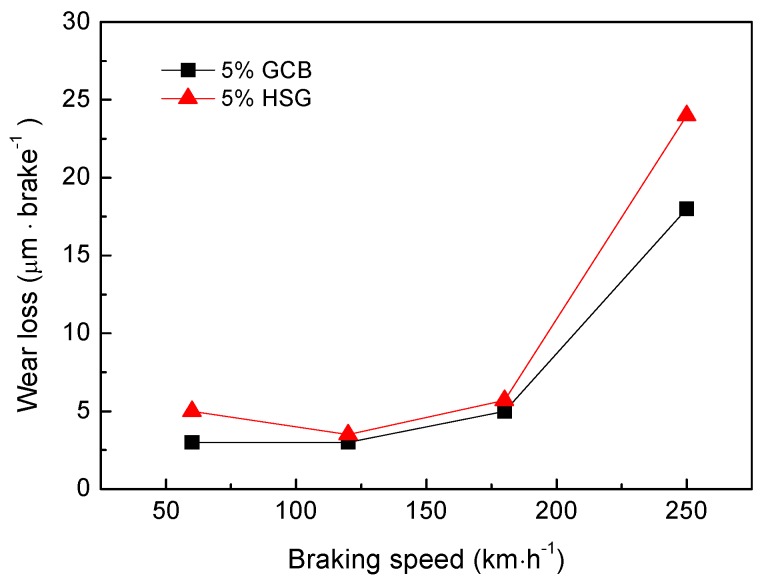
Effect of different lubricating phase on wear loss of materials.

Figure 11 shows the effect of different lubricating phase on wear loss of materials under the same mass fraction (5 wt%). The sample of 5% HSG exhibits a higher wear loss at the same braking speed. With the increase of the braking speed, the wear loss of the materials shows a basically consistent variation tendency. When the braking speed is lower than 180 km·h^−1^, the wear loss of the material is low and the difference of the wear loss of each speed is not obvious, but when the speed is higher than 180 km·h^−1^, the wear loss of the material increases abruptly. 

The wear of the friction material is caused by the damage of the friction surface film during the braking process. The sample with 5 wt% granulated carbon black as the lubricating phase has a lower wear loss due to its good interfacial adhesion to the matrix, lower porosity, higher strength, and relatively intact friction surface film. When the braking speed increases, the friction surface temperature increases continuously, while the higher temperature decreases the strength of the matrix, which causes the accelerated destruction of the friction surface film. It can be seen from Figure 9 that the friction surface temperature increases with the increase of the braking speed. When the speed is lower than 180 km·h^−1^, the temperature rise is gentle, but when the speed is higher than 180 km·h^−1^, the friction surface temperature increases rapidly, which increases the damage degree of friction film. This explains the reason why the wear loss of the material does not change much at low speed and the abrupt increase of the wear loss exceeds 180 km·h^−1^.

## 4. Conclusions

In this paper, Cu–Fe based friction materials with flake graphite, granulated carbon black, and high-strength graphite as lubricating phase were prepared by powder metallurgy method. Meanwhile, the effects of different types and mass fraction of lubricating phase on the microstructure, mechanical properties, and tribological properties were investigated. The conclusions are drawn as follows:

(1) The mass fraction of granulated carbon black was 5 wt%, and it was easy to form a good interface with the matrix, but the interface was prone to pores and cracks when its mass fraction was 10 wt%. The interface between the high-strength graphite and matrix was also poor.
(2) The bending strength and compressive strength properties of the composites increased with increasing in the mass fraction of granulated carbon black and reached the maximum of 40 MPa and 70 MPa at 5 wt% granulated carbon black, after which bending strength and compressive strength all decreased. The friction coefficient and the wear loss of the materials initially decreased as the mass fraction of granulated carbon black increased and obtained minimum of 0.436 and 0.145 mm when the mass fraction of granulated carbon black was 5 wt%, then ascended.
(3) Compared with the sample with 5 wt% high-strength graphite as lubricating phase, the sample with 5 wt% granulated carbon black as the lubricating phase had better sintering performance and mechanical properties, lower friction coefficient, and wear loss.

## Figures and Tables

**Table 1:** Compositions of Cu–Fe based friction materials (wt%).

Samples	Cu (<75 μm)	Fe (<75 μm)	Fe-Cr (<200 μm)	Flake Graphite (100–600 μm)	Granulated Carbon Black (200–500 μm)	High-Strength Graphite (200–500 μm)
0% GCB	52	23	10	15	0	0
5% GCB	52	23	10	10	5	0
10% GCB	52	23	10	5	10	0
5% HSG	52	23	10	10	0	5

**Table 2:** Sintering and mechanical properties with different lubricating phase.

Samples	Density (g·cm^−3^)	Porosity (%)	Bending Strength (MPa)	Compressive Strength (MPa)
0% GCB	4.76	9.64	32	52
5% GCB	5.10	6.89	40	70
10% GCB	5.02	8.52	32	62
5% HSG	4.93	9.21	30	59
